# Comparative development of the serotonin- and FMRFamide-immunoreactive components of the nervous system in two distantly related ribbon worm species (Nemertea, Spiralia)

**DOI:** 10.3389/fnins.2024.1375208

**Published:** 2024-03-22

**Authors:** Jörn von Döhren

**Affiliations:** Bonn Institute of Organismic Biology (BIOB), Animal Biodiversity Section, University of Bonn, Bonn, Germany

**Keywords:** larva, development, nervous system, FMRFamide, serotonin, synapsin, Nemertea, Lophotrochozoa

## Abstract

**Introduction:**

Neurodevelopment in larval stages of non-model organisms, with a focus on the serotonin- and FMRFamide-immunoreactive components, has been in the focus of research in the recent past. However, some taxonomic groups remain understudied. Nemertea (ribbon worms) represent such an understudied clade with only few reports on nervous system development mostly from phylogenetically or developmentally derived species. It would be insightful to explore neurodevelopment in additional species to be able to document the diversity and deduce common patterns to trace the evolution of nervous system development.

**Methods:**

Fluorescent immunohistochemical labeling with polyclonal primary antibodies against serotonin and FMRF-amide and a monoclonal antibody against synapsin performed on series of fixed larval stages of two nemertean species *Cephalothrix rufifrons* (Archinemertea, Palaeonemertea) and *Emplectonema gracile* (Monostilifera, Hoplonemertea) were analyzed with confocal laser scanning microscopy.

**Results:**

This contribution gives detailed accounts on the development of the serotonin- and FMRFamide-immunoreactive subsets of the nervous system in two nemertean species from the first appearance of the respective signals. Additionally, data on synapsin-like immunoreactivity illustrates the general structure of neuropil components. Events common to both investigated species are the appearance of serotonin-like immunoreactive signals before the appearance of FMRF-like immunoreactive signals and the strict progression of the development of the lateral nerve cords from the anteriorly located, ring-shaped brain toward the posterior pole of the larva. Notable differences are (1) the proboscis nervous system that is developing much earlier in investigated larval stages of *E. gracile* and (2) distinct early, but apparently transient, serotonergic neurons on the frontal and caudal pole of the larva in *E. gracile* that seem to be absent in *C. rufifrons*.

**Discussion:**

According to the results from this investigation and in line with previously published accounts on nervous system development, the hypothetical last common ancestor of Nemertea had a ring-shaped brain arranged around the proboscis opening, from which a pair of ventro-lateral nerve cords develops in anterior to posterior progression. Early frontal and caudal serotonergic neurons that later degenerate or cease to express serotonin are an ancestral character of Nemertea that they share with several other spiralian clades.

## 1 Introduction

Morphological Evolutionary Development (MorphoEvoDevo), i.e., comparative investigation of the development of organ systems or subsets thereof, has greatly advanced our knowledge on the evolution and development of these organ systems and has lent independent support to the new animal phylogeny that has mainly been based on the analysis of molecular data (Wanninger, 2007, 2015). Especially in the spiralian (= lophotrochozoan) clades of Mollusca and Annelida, results from a number of investigations have resolved conflicts between molecular phylogenies, e.g., the secondary loss of segmentation in Echiura (McHugh, 1997; Hessling, 2002; Hessling and Westheide, 2002). In other cases, classical morphological hypotheses were supported by the new data, e.g., the sister-group relationship of Mollusca and Entoprocta (Bartolomaeus, 1993; Haszprunar, 1996; Friedrich et al., 2002; Voronezhskaya et al., 2002; Wanninger et al., 2007).

Immunohistochemistry with antibodies against serotonin and the FMRFamide neuropeptide group have been most widely employed in investigations of nervous system development. Accumulation of data for many spiralian species open up the opportunity to reconstruct the ancestral traits of the respective clades and thus consolidate the existing hypotheses on phylogeny and evolution of Spiralia and the development and homology of their organ systems. Unfortunately, some of the clades that are most unstable in phylogenetic analyses are also poorly represented in the MorphoEvoDevo-datasets.

**FIGURE 1:**
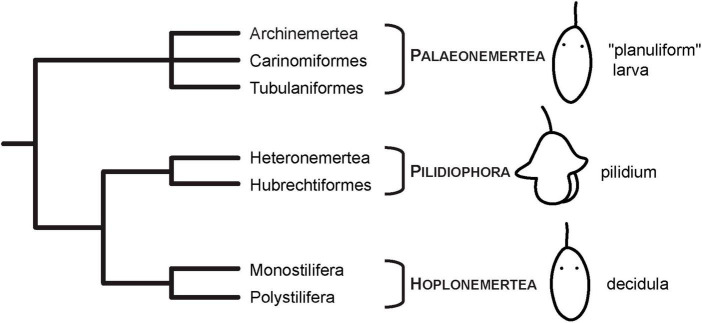
Current phylogenetic hypothesis and larval types of Nemertea. Phylogeny modified after Andrade et al. (2014) and Kvist et al. (2014). Images of larvae modified from Hindinger et al. (2013), under the terms of CC-BY 2.0.

One of these clades are Nemertea (ribbon worms), a group of unsegmented, mainly predatory, worm-shaped marine animals that develop via clade-specific larval types (Maslakova, 2010b; Maslakova and Hiebert, 2014; von Döhren, 2015). The most well-known larval type is the pilidium larva that is characteristic of the clade Pilidiophora (Thollesson and Norenburg, 2003; Andrade et al., 2014; Figure 1). Several investigations exist that report the development of musculature and/or subsets of the nervous system by means of fluorescent or immuno-fluorescent (IF) staining and analysis by confocal laser scanning microscopy (CLSM) (Hay-Schmidt, 1990; Maslakova, 2010a; von Döhren, 2011; Hindinger et al., 2013). However, since the pilidium larva is derived within Nemertea, it cannot be used for reconstruction of ancestral traits in Nemertea (Maslakova, 2010b; Maslakova and Hiebert, 2014; Beckers and von Döhren, 2015; von Döhren, 2015; Figure 1). Reconstruction of ancestral traits in the development of Nemertea needs a detailed look at the larvae of the remaining nemertean clades, Palaeonemertea and Hoplonemertea. In contrast to the traditional view of these nemertean clades showing direct development, it is now clear, that there is a larval stage that possesses various morphological structures that are confined to this developmental phase. Palaeonemertean species develop via a pelagic, relatively unspecific, and hence termed planuliform larva that has occasionally been related to a trochophore-type larva (Figure 1). Several species have larval ocelli that are not found in adult specimens (von Döhren, 2016a; von Döhren and Bartolomaeus, 2018). The pelagic larva of hoplonemertean species is characterized by the shedding of its first generation of epidermis cells and has therefore been termed decidula (Maslakova, 2010b; Maslakova and Hiebert, 2014; Figure 1). Whereas for larvae of Hoplonemertea, some investigations on nervous system development have been published, the reports on the larvae of the basally branching Palaeonemertea remain relatively scarce. Furthermore, only few investigations that focus on the developmental sequence starting with the earliest stages of development exist.

This article aims at contributing to the knowledge on the development of the nervous system components that are immunoreactive against serotonin, FMRFamide, and synapsin antibodies in a hoplonemertean and a paleonemertean species, from the earliest developmental stages. Analysis of IF with CLSM allows acquisition of detailed comparative data. The investigated species develop via larval stages that are less derived than the larva of Pilidiophora and therefore more suitable to reconstruct ancestral states of nemertean nervous system development.

## 2 Materials and methods

### 2.1 Species and collecting sites

Sexually mature females and males of *Emplectonema gracile* (Johnston, 1837) (Monostilifera, Hoplonemertea) (RRID:*NCBITaxon_6230*) and *Cephalothrix rufifrons* (Johnston, 1837) (Archinemertea, Palaeonemertea) were collected during visits at the Station de Biologie Marine de Concarneau (Dpt. Finistère, France). *E. gracile* was found under stones in the intertidal zone of the rocky shore near Le Cabellou (Dpt. Finistère, France) at low-tide between July and August of the years 2012 and 2016. *C. rufifrons* was found in entangled masses of several individuals in sand underneath stones on a sandy beach with boulders near Pointe de Jument (Dpt. Finistère, France) in April 2016. Animals were transferred to the Institute of Evolutionary Biology and Ecology of the University of Bonn (Germany), where *E. gracile* individuals were kept together in plastic boxes with lid (approximately 500 ml capacity) with some stones at 18°C. Individuals of *C. rufifrons* were kept in glass petri-dishes filled with sand from the collecting site and artificial sea water at 12°C (*C. rufifrons*). Water was changed twice per week.

### 2.2 Obtaining and rearing larvae

In both species, both sexes were kept together and the containers were checked daily for spawned clutches. *C. rufifrons* was furthermore conditioned to spawn by keeping it at around 8°C overnight and replacing the water with 12°C warm artificial seawater in the morning. Both species were kept in 1.5-L capacity plastic boxes filled with 1 L of conditioned artificial seawater. Water was changed every 2–3 days by reverse filtering through a 50 μm-mesh size gauze. Copepodid stages of unidentified Copepods and nauplii of *Artemia franciscana* Kellog, 1906 (Anostraca, Branchiopoda) (RRID:*NCBITaxon_6661*) were offered as food to the larvae of *E. gracile*. However, the offered food organisms were either not appropriate or not offered in sufficient quantities to ensure continuous development past 8 days after hatching. *C. rufifrons* larvae were initially fed every 2–3 days with unfertilized oocytes of the heteronemertean species *Riseriellus occultus* Rogers, Junoy, Gibson and Thorpe, 1993 (Heteronemertea, Pilidiophora). After 21 days, unfertilized oocytes of the annelid species *Platynereis dumerilii* (Audouin and Milne Edwards, 1833) (Phyllodocida, Errantia) (RRID:*NCBITaxon_6359*) were provided as food.

### 2.3 Sampling and fixation of larval stages

Developmental stages of two different batches of larvae (2012 and 2016) of *E. gracile* were sampled every day from 1 day after oviposition (dpo) to 6 dpo and at 8 dpo. Due to starvation of the larvae, no more samplings were attempted after 8 dpo. In *C. rufifrons*, investigated specimens were obtained from two batches of larvae. Samples were obtained every day from 3 dpo to 5 dpo, and afterward at 7 dpo, 9 dpo, 11 dpo, 14 dpo, 21 dpo, 28 dpo, and 42 dpo. At the last timepoint of sampling (42 dpo), the number of larvae had diminished to such extent that additional sampling was not considered reasonable. Prior to fixation, larvae were washed with three changes of ultra-filtered artificial sea water. From 2 dpo onward in *E. gracile* and 4 dpo onward in *C. rufifrons*, larvae were anesthetized with ultra-filtered (0.2 μm) 3.5% MgCl_2_ (prepared from equal amounts of 7% MgCl_2_ solution in distilled water and artificial sea water) at room temperature for 15 min. Subsequently, larvae were fixed in ultra-filtered, 4% para-formaldehyde solution (PFA, freshly prepared from one part of a 16% PFA solution and three parts of artificial sea water) in glass bowls (approximately 4 ml capacity) at 18–20°C for 30 min (to 45 min for *C. rufifrons* from 14 dpo onward). Afterward, the larvae were washed three times for 10 min each in 0.1 M phosphate buffer saline (PBS) at room temperature. Subsequently, the larvae were stored at 4–8°C in the same buffer but with 0.01% (w/v) NaN_3_ added to prevent microbial growth.

### 2.4 Immunohistochemistry, fluorescent labeling, and mounting

Some larvae of both species were treated with Image-iT signal enhancer (ThermoFisher, I36933) according to the manufacturer’s protocol for a minimum of 1 h at room temperature. Larvae of *C. rufifrons* 21 dpo and older were postfixed in 4% PFA in PBS for 10–15 min.

All larvae were washed in three changes of PBT (PBS with 0.1%–0.3% v/v Triton X-100 added) for 10 min each prior to blocking in PBT with 5%–10% normal goat serum (NGS) for 1–2 h at room-temperature. Subsequently, the larvae were incubated with the primary antibodies diluted in 0.1%–0.3% PBT at 18°C on a rocking table overnight. Polyclonal primary antibodies applied were against serotonin produced in rabbit (Sigma-Aldrich Cat# S5545, RRID:*AB_477522* for *E. gracile* or ImmunoStar Cat# 20080, *RRID:AB_572263* for *C. rufifrons* and *E. gracile*) at a dilution of 1:1,000, FMRFamide produced in rabbit (ImmunoStar Cat# 20091, RRID:*AB_572232* for *E. gracile* or Abcam, ab10352-50 for *C. rufifrons*) at a dilution of 1:750 or 1:1,000. Against synapsin, anti SYNORF1 (= 3C11, DSHB Cat# CPTC-Cd248-3C11, RRID:AB_1553491, University of Iowa) produced in mouse at a dilution of 1:10 in *E. gracile* and 1:50 in *C. rufifrons* were used. Each primary antibody was diluted from a stock solution of 1 mg/ml. The antibody 3C11 (anti SYNORF1) was deposited to the DSHB by E. Buchner (DSHB Hybridoma Product 3C11; Klagges et al., 1996). After incubation with primary antibodies, specimens were washed in quickly in three changes of 0.1%–0.3% PBT followed by three changes of the same buffer for 10 min each at room temperature.

Secondary antibodies were subsequently applied in 0.1% PBT. Against rabbit antibodies, goat anti rabbit conjugated to Alexa Fluor 488 (ThermoFisher, A11008), Alexa Fluor 633 (ThermoFisher, A21071) or Cy2 (Immunostar, 111-225-144) at a dilution of 1:100 or Alexa Fluor 568 (ThermoFisher, A11011) at a dilution of 1:200 were used. As secondary antibodies against anti-synapsin, goat anti mouse conjugated to either Alexa Fluor 568 (ThermoFisher, A11004) or Cy5 (Immunostar, 115-175-146) at a dilution of 1:100 were used. All secondary antibody were diluted from a stock solution of 1 mg/ml. The specimens were incubated with the secondary antibodies for 2–2.5 h at room temperature and subsequently washed three times for 10 min each in 0.1% PBT. Details on variation in the immunostaining protocol (mainly due to different sizes of the larvae) are listed in Supplementary Table 1.

After the final washing step, the larvae were fastened to cover slips coated with 0.01% poly-L-lysine-hydrobromide. The preparations were dehydrated using a graded series of isopropanol (70%, 85%, 95%, twice 100%) for 1 min each followed by three changes of Murray’s Clear (= BABB: one part of benzyl alcohol mixed with two parts of benzyl benzoate) for 10 min each. The preparations were then mounted in Murray’s Clear using small pieces of modeling clay as spacers between glass slide and cover slip and sealing the gaps with nail polish.

### 2.5 Confocal laser scanning microscopy and image processing

To follow the development of the nervous system, a minimum of four larvae per developmental stage were examined. For image acquisition a Leica TCS/SPE confocal laser scanning system mounted on a Leica DM 2500 microscope was used. Alexa Fluor 488 and Cy2 were excited with the 488 nm, Alexa Fluor 568 with the 532 nm, and Alexa Fluor 633 and Cy5 with the 635 nm laser lines. All images were recorded with a 40× dry objective (NA 0.75) with the pinhole diameter set to one airy disc size. Stacks of images were recorded with a virtual thickness of 0.88–1.01 μm at 1,024 × 1,024 pixels and 8-bit image depth, except for 21 dpo and 42 dpo *C. rufifrons* larvae that were recorded at 12-bit image depth. Z-projections and image adjustments were made in ImageJ version 1.54h (RRID:*SCR_003070*) of the Fiji distribution package Fiji (RRID:*SCR_002285*) (Schindelin et al., 2012; Schneider et al., 2012). Details on image processing are listed in Supplementary Table 1. Image plates were mounted and labeled with Adobe Illustrator CS6 (Adobe Systems Inc., RRID:*SCR_010279*).

## 3 Results

### 3.1 *Emplectonema gracile* (Monostilifera, Hoplonemertea)

#### 3.1.1 General development

**FIGURE 2:**
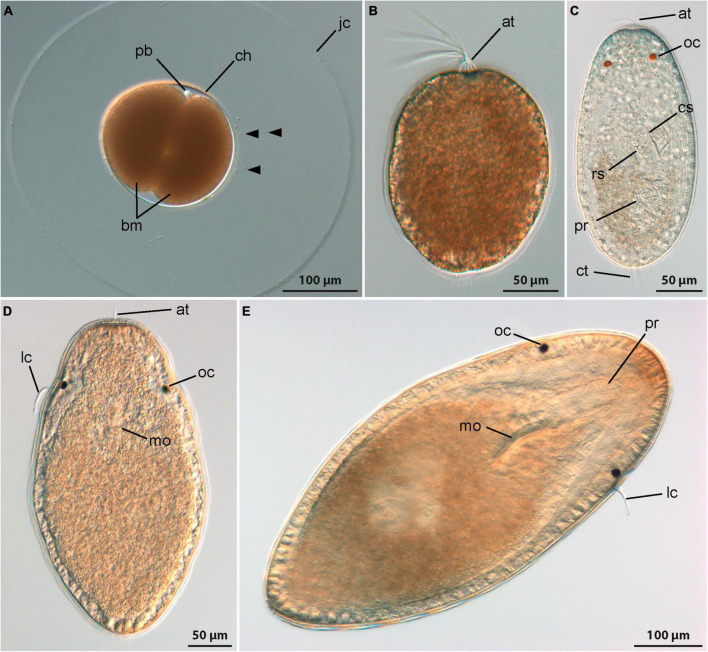
Development (DIC, all images from living specimens); **(A–C)** *Emplectonema gracile* and **(D,E)** *Cephalothrix rufifrons*. **(A)** Two-cell stage, approximately 12 h after oviposition (lateral view, apical to top-right corner), note excess sperm cells caught in jelly coat (arrowheads); **(B)** young larva shortly after hatching, approximately 1 day after oviposition (apical is up); **(C)** older larva 7 days after oviposition (dorsal view, apical is up); **(D)** young larva at 11 days after oviposition (ventral view, apical is up); **(E)** older larva 42 days after oviposition (ventral view, apical to top-right corner). at, apical tuft; bm, blastomeres; ch, chorion; cs, central stylet; ct, caudal tuft; jc, jelly coat; lc, lateral cirrus; mo, mouth opening; oc, ocellus; pb, polar bodies; pr, proboscis; rs, reserve stylet.

*Emplectonema gracile* spawns lightly brown colored eggs with a diameter of approximately 150 μm that are individually enclosed in a gelatinous envelope (Figure 2A, jc). After spawning, the embryos develop inside the chorion for about a day at 18°C until they hatch. For monostiliferan species, a staging system has been published (Hiebert et al., 2010). This staging system relies on data obtained with phalloidin staining of fibrillar actin and landmarks from external appearance. Since data on f-actin are not reported in this account, general landmarks, such as changes in body shape, the formation of the apical pit with a tuft of elongated cilia, and the emergence of a caudal tuft of cilia are herein used to relate the published staging system to the larval development of *E. gracile*. The first stage is called invagination stage and is characterized by a larva with a spheroid body shape. In the evenly ciliated larvae, an apical pit and an anterior mouth opening are present while the apical tuft of elongated cilia emanating from the apical pit has only formed by the end of the invagination stage. In *E. gracile*, larvae hatch at 1 dpo in the early invagination stage and by 2 dpo the late invagination stage is reached (Figure 2B). However, at 2 dpo, most larvae have already transited to the subsequent stage, called rudiment stage. The larvae attain an elongated shape with a bluntly tapering caudal end. The proboscis rudiment is formed initially as a bipartite structure inside the larvae and a pair of eyes appears on the dorso-lateral sides of the body. During the late rudiment stage, formation of a tuft of elongated cilia at the caudal end and the differentiation of the middle portion of the proboscis with the formation of the stylet apparatus mark the transition to the vermicular stage, that is reached at 3 dpo (Figure 2C). In the vermicular stage, the body has further elongated, also in the precerebral area, and becomes worm-like. The mouth opening is still present on the ventral side of the larva, closely posterior to the brain ring. In *E. gracile*, its definite closure and the subsequent opening of the stomodeum into the rhynchodeum is assumed to take place later during development. However, further development could not be followed in this study due to the inability to provide appropriate food to ensure development further than 8 dpo.

#### 3.1.2 Development of FMRFamide-like immunoreactivity

**FIGURE 3:**
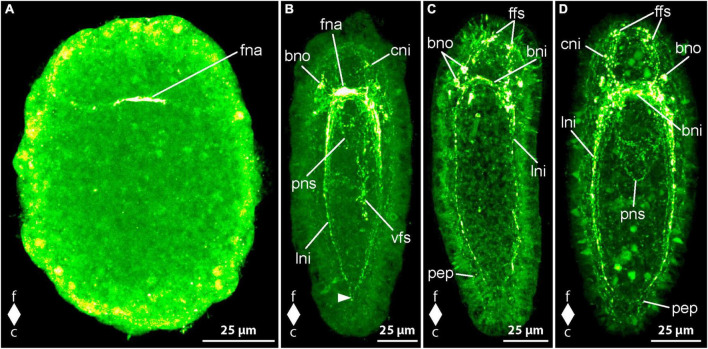
*Emplectonema gracile* larvae, FMRFamide-like immunoreactivity, maximum projection, frontal faces up in all images. **(A)** 1 day after oviposition, dorsal view (*z* ≈ 49 μm, γ = 1.5); **(B)** 3 days after oviposition, dorso-lateral view (*z* ≈ 48 μm, γ = 0.6), note the caudal connection of the lateral longitudinal nerve cords by the anal commissure (arrowhead); **(C)** 5 days after oviposition, ventral view (*z* ≈ 59 μm, γ = 1.2); **(D)** 6 days after oviposition, ventral view (*z* ≈ 48 μm, γ = 0.7). bni, brain ring neurites; bno, brain ring neuron; c, caudal; cni, cephalic neurites; f, frontal; ffs, frontal RFa-lir signals; fna, fronto-dorsal neurite aggregation; lni, longitudinal nerve cord neurites; pep, peripheral epidermal plexus; pns, proboscis nervous system; vfs, ventral RFa-lir signal aggregation.

The earliest stages that show FMRFamide-like immunoreactivity (RFa-lir) signals are newly hatched larvae about 1 dpo. The most conspicuous signal seen in all larvae is a short, transversal, neurite-like signal dorsally in the frontal part of the body (Figure 3A, fna). A pair of short signals that resemble neurites frontal on the level of the dorsal neurite-like signal but on the ventral side of the larva is clearly seen in some of the allegedly more advanced specimens examined. A connection between the dorsal and the ventral signals is not evident, but it is likely that these signals represent the first neurite-like signals of the developing brain ring.

In larvae at 2 dpo, the brain is seen as one to two neurite-like signals forming a ring that is oriented perpendicularly to the longitudinal body axis near the frontal end of the larva. In a medio-dorsal position, a conspicuous signal that might represent an aggregation of neurites is visible in the position where the first transversal dorsal neurite-like signal was found in the larvae at 1 dpo. At most one pair of neuron-like signals associated with the ventral portion of the brain ring neurite-like signals is found. Each signal is located caudally and ventro-laterally of the brain ring neurite-like signals, near their transition to the lateral nerve cords on both sides of the body. On the dorsal side of the body there are few more paired neuron-like signals associated with the brain ring neurites detectable. These neuron-like signals are often slightly larger than the neuron-like signals on the ventral side and amount to a maximum of two to three well visible and some additional, very weak signals not consistently found in every investigated larva. The conspicuous dorsal brain ring associated neuron-like signals are connected to the dorso-median neurite aggregation-like signal by fine neurite-like processes. In very few larvae a weak neuron-like signal is detected frontal of and near the dorsal section of the brain ring neurite-like signals. It seems to possess an apical process that extends frontally toward the vicinity of the apical pit. Along the ventro-lateral sides of the body the first neurite-like signals of the lateral nerve cords are visible. They comprise one to two signals extending from the brain ring neurite-like signals caudally to about two thirds of the body length. In the allegedly more advanced stages examined, the neurite-like signals of the lateral nerve cords amount to up to three signals extending almost to the caudal end of the larva. In many of these larvae an inconspicuous, so-called anal commissure, represented by an arcuate dotted line connects the lateral nerve cord signals at their caudal ends. The developing proboscis, that consists of two distinguishable parts at this stage of development shows comparably strong RFa-lir signals in the transition zone between the anterior and the posterior portions in more advanced larvae.

In 3 dpo larvae, the brain ring consists of two neurite-like RFa-lir signals that, in the ventral portion of the brain ring, have become brighter than in earlier stages. The neurite-like signals in the dorsal portion remain largely unchanged in intensity and number and the neurite aggregation-like signal is still the most obvious signal in this part of the brain (Figure 3B, fna). The number of neuron-like signals has not obviously changed, although the signals, especially those of the dorsal brain neurons, have become more evident in some specimens. In the precerebral region of several specimens, there are some weak, neurite-like signals observed that extend from the brain region toward the frontal tip of the body. These signals might represent the first neurite-like signals of the so-called cephalic nerves (Figure 3B, cni). The lateral nerve cords comprise one to two bundles of neurite-like signals consisting of few neurite-like signals. The signals of the lateral nerve cords extend almost to the caudal end of the body and a so-called anal commissure is seen in almost all investigated specimens, albeit its signal is not very strong in most larvae (Figure 3B, arrowhead). The developing proboscis apparatus still shows comparably strong RFa-lir signals in the anterior region of its posterior portion. However, the signals seem to be relatively less intensive than in larvae at 2 dpo. In more advanced stages at this age, the proboscis begins to become tripartite, the former anterior portion further having divided into an anterior and a middle portion. In many larvae at this advanced developmental stage, there is a weak ring-shaped neurite like signal visible in the anterior portion of the proboscis (Figure 3B, pns). An aggregation of conspicuous RFa-lir signals that are very variable in size and shape is seen in the epidermis on the ventral side of the larva around the mouth opening and extends caudally up to about two thirds of the total body length (Figure 3B, vfs). From their shape, it cannot be stated clearly if these signals represent neurons or neurites.

Larve at 4 dpo show an increase in the number and intensity of neurite-like signals in the ventral portion of the brain ring to up to three bundles in some examined specimens. The dorsal portion of the brain ring neurite-like and the neuron-like signals do not show considerable development compared to earlier developmental stages. In the precerebral region, frontal of the brain ring neurite-like signals of so-called cephalic nerves are observed in all specimens. In some of these, there are several dot-shaped epidermal signals arranged in the vicinity of the apical pit. These signals might represent apical parts of epidermal sensory cells. The signals of the lateral nerve cords comprise two bundles of neurite-like signals in more larvae compared to younger larvae and the signal have become more evident. In about half of the examined specimens, a pair of weak, laterally located neuron-like signal is observed along each side of the lateral nerve cords frontally at about a quarter of their total extension. The developing proboscis shows neurite plexus-like signals in its anterior portion that are mostly dim and not seen in all specimens. In the middle portion of the proboscis a ring-shaped neurite-like signal is seen in almost all of the larvae, although in most, it is comparably weak. In the posterior portion, mostly dim RFa-like signals are only present in a few larvae. Comparably weak neurite plexus-like signals can be seen around the esophagus, whereas the ventral epidermal signals in the vicinity of the mouth opening and further caudally cannot be detected in larvae at 4 dpo.

At 5 dpo, the dorsal portion of the brain ring neurite like signals consists of three bundles of which the caudal-most bundle shows the strongest signals. The remaining neurite-like signals of the brain ring have not developed further considerably, the number of ventral brain ring neuron-like signals being unchanged and number the dorsal brain ring neuron-like signals possibly having increased by few pairs of very inconspicuous signals. Whereas the neurite-like signals of the so-called cephalic nerves have remained largely unchanged, the number of epidermal frontal signals in the vicinity of the apical pit has increased compared to earlier developmental stages (Figure 3C, ffs). In the lateral nerve cords there is also little indication of changes in RFa-lir signals. In the proboscis, the intensity of the signals seems to increase from the posterior to the anterior portion, with neurite-like signals being conspicuous in most larvae in the anterior portion but in the posterior portion only in few investigated specimens. The peripheral epidermal plexus is represented by neurite plexus-like signals found in all parts of the epidermis in the majority of larvae (Figure 3C, pep). In only a few larvae these signals are brighter. The signals of the esophageal nervous system show no obvious change in intensity compared to larvae at 4 dpo.

At 6 dpo, the majority of nervous system-like signals shows no obvious change. Exceptions include the number of neurite-like signals in the brain ring, that has increased in its dorsal part to one to two bundles, each consisting of few closely apposed neurite-like signals. The number of neuron-like, brain-associated signals has increased in the ventral portion by one additional pair, each signal being located caudally on either side of the brain ring, lateral to the lateral nerve cord neurite-like signals (Figure 3D, bno). The three bundles of neurite-like signals of the lateral nerve cords have become stronger and the so-called anal commissure is clearly visible in all examined larvae. The pair of faint neuron-like signals next to lateral nerve cord neurite bundles seem to possess an apical process extending to the epidermis in some larvae. In the proboscis, the neurite-like signals of all three portions can be detected in all larvae. In the anterior portion the signals are conspicuous in all larvae, in the middle portion the signals are obvious in most larvae and in the posterior portion several larvae show strong neurite-like signals (Figure 3D, pns). The signals of the peripheral epidermal plexus and the signals of the plexus around the esophagus have become stronger in more larvae compared to the preceding developmental stage. The majority of larvae at 8 dpo are similar to larvae at 6 dpo in development of nervous system like signals. Only a few larvae show changes in observed signals that indicate further development. The observed developmental arrest in most larvae at 8 dpo might be indicative of beginning starvation of the larvae due to absence of appropriate food. In the very few larvae that show developmental progress the number of obvious neuron-like signals associated with the dorsal part of the brain ring has increased to up to four pairs on each side of the dorsal midline. Two pairs are located frontal and two pairs caudal of the brain ring neurite-like signals. Progress is also observable in the signals of the peripheral epidermal plexus that are clearly seen in all of the respective larvae. The remaining components of the RFa-lir nervous system seem to remain unchanged compared to earlier investigated developmental stages.

#### 3.1.3 Development of serotonin-like immunoreactivity

**FIGURE 4:**
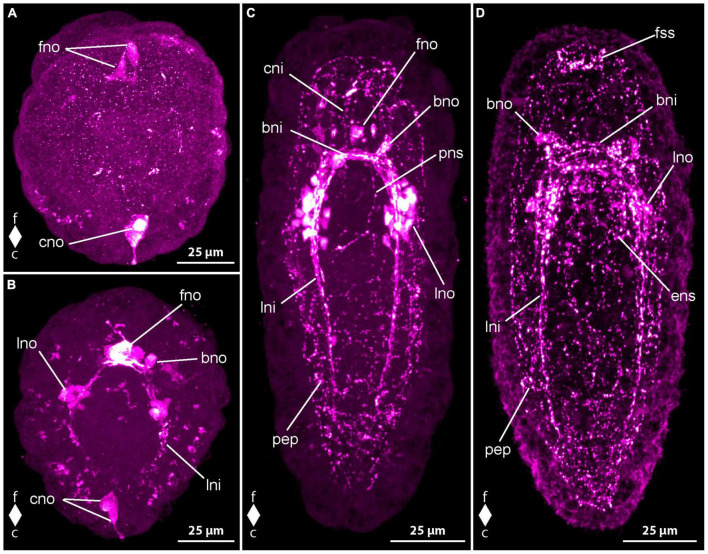
*Emplectonema gracile* larvae, serotonin-like immunoreactivity, maximum projection, frontal faces up in all images. **(A)** 1 day after oviposition, dorsal view (*z* ≈ 84 μm, γ = 1.5); **(B)** 2 days after oviposition, dorsal view (*z* ≈ 66 μm, γ = 0.8); **(C)** 3 days after oviposition, dorsal view (*z* ≈ 56 μm, γ = 0.8); **(D)** 6 days after oviposition, ventral view, (*z* ≈ 43 μm, γ = 1.1). bni, brain ring neurites; bno, brain ring neuron; c, caudal; cni, cephalic neurites; cso, caudal 5HT-lir neuron; esn, esophageal nervous system; f, frontal; fso, frontal 5HT-lir neuron; fss, frontal 5HT-lir signals; lni, longitudinal nerve cord neurites; lno, longitudinal nerve cord neuron; pep, peripheral epidermal plexus; pns, proboscis nervous system.

In newly hatched larvae, 1 dpo, there is considerable variation in the number of observed serotonin-like immunoreactivity (5HT-lir) signals. This variation hints at a very fast development during the earliest phase of development. Most early larvae show two comparably large, frontal, neuron-like signals that possess an apical process that extends to the vicinity of the apical pit (Figure 4A, fno). In some larvae there is only one such neuron-like signals present. In most larvae with two frontal neuron-like signals, there is a neuron-like signal present at the caudal pole of the larva that also possesses a slender process to the epidermis (Figure 4A, cno). One frontal neuron-like signal without apical processes is seen in direct apposition to the one or two frontal neuron-like signals with apical process. Basally, a variable number, but never numerous, neurite-like signals emanate from the frontal group of neuron-like signals. In very few specimens a second frontal neuron-like signal without apical process is grouped with the frontal aggregation of neuron like signals. More advanced stages show neuron- and neurite-like signals on the lateral sides of the body at about half of its length. The lateral signals are likely the first signals of the lateral nerve cords.

In larvae at 2 dpo, the first signals of the brain ring and the lateral nerve cords are visible. The brain ring comprises up to two individual neurite-like signals in its ventral part and up to one signal in the dorsal portion. Up to one pair of neuron-like signals are detectable on the fronto-lateral sides of the brain ring. Dorsally, up to two pairs of neuron-like signals are found associated with the brain ring (Figure 4B, bno). They are located on the caudal side on both sides of the median line of the brain ring and each one seems to be connected to the median portion of the brain ring by a weak neurite-like signal. Frontal of the brain ring in a few larvae, up to four larger, subepidermal neuron-like signals are present, some with an apical process to the vicinity of the apical pit (Figure 4B, fno). In some specimens, the perikarya-like signals seem to sit on the neurite-like signals of the dorsal part of the brain ring. In others, a neurite-like signal or a thin bundle thereof extends from the basis of the neuron-like signal to each of the lateral sides of the dorsal brain ring neurite-like signals. In a few, allegedly more advanced larvae, there are a few weak, neurite like signals extending more superficially from the brain ring region longitudinally toward the frontal end of the body. These signals are interpreted as neurites of the so-called cephalic nerves. The signals of the lateral nerve cord are one to two neurite-like signals that extend caudally to about half to three quarters of the total body length (Figure 4B, lni). Shortly caudal of the transition of the brain ring to the lateral nerve cord signals, there is a group of several neuron-like signals that surround the neurite like signals of the lateral nerve cords on both sides of the body (Figure 4B, lno). The number of these neuron-like signals is very variable, so that from 4 to 10 can be counted on each side. A number of five to six signals is seen in the majority of larvae. In some more advanced specimens, there is a weak neurite-like signal in the developing proboscis, which is bipartite at this developmental stage. The signal is paired and runs in longitudinal orientation along both sides of the anterior portion of the proboscis. Neurite-like signals in the epidermis on all sides of the body, most likely representing the 5HT-lir component of the peripheral epidermal plexus are seen in most larvae. However, these signals are absent or inconspicuous in younger developmental stages. In a few of these earlier stages, the neuron-like signal at the caudal end of the larva is still present (Figure 4B, cno).

At 3 dpo, the number of brain ring neurite-like signals has increased to three to four in the ventral part and up to two in the dorsal part. A second pair of neuron-like signals on the lateral side of the brain ring, is showing on the same level as the brain ring neurite like signals, but further distally. Dorsally, the number of brain ring neuron-like signals has not obviously increased. Cephalic nerve neurite-like signals are detectable in many larvae (Figure 4C, cni). The most conspicuous of these is a bow of neurite-like signals that extends on the lateral sides of the precerebral region to be joined at the frontal tip in the vicinity of the apical pit. The neurite-like signals of the lateral nerve cords have increased in length and in number to three or four. They reach caudally to almost the caudal end of the body (Figure 4C, lni). In most of the larvae, there is a bow-shaped caudal connection of the neurite-like signals, the so-called anal commissure. The proboscis neurite-like signals in the now tripartite proboscis become stronger and elongate frontally to the lateral brain ring neurite-like signals in some specimens (Figure 4C, pns). On the ventral side, several neurite-like and neuron-like signals surround the mouth opening and the distal portion of the esophagus in a plexus-like fashion in some more advanced larvae. Neurite-like signals of the peripheral epidermal plexus are evident in all examined larvae (Figure 4C, pep). The caudal neuron-like signal has disappeared from all investigated specimens. Likewise, the frontal, subepidermal, neuron-like signals are only found in very few larvae. In these larvae, at most three signals, most of them without apical process to the vicinity of the apical pit are detectable (Figure 4C, fno). In the other examined specimens, the frontal signals have been replaced by a low number of small epidermal signals, that are found in the vicinity of the apical pit. Some of them seem to be connected to the neurite-like signals of the cephalic nerves. These epidermal signals might represent portions of epidermal sensory cells.

The neurite-like signals of the brain ring have further increased in number in larvae at 4 dpo, so that they form two conspicuous and a few less evident bundles in the ventral part and two slightly weaker bundles in the dorsal part of the brain ring. The number and intensity of neuron-like signals lateral to the brain ring neurite-like signals has not obviously changed but in the dorsal portion of the brain ring, an additional pair of neurons, frontal and lateral of the neurite-like signals is present in most specimens. The lateral nerve cord neurite-like signals forms two to three conspicuous bundles and an anal commissure is evident in all specimens. The number of neurons associated with the frontal-most portion of the lateral nerve cords has not obviously changed in number although a slightly higher number of neurons shows weaker signals than in larvae at 3 dpo. Frontal of the brain, the neurite-like signals of the cephalic nerves have not markedly increased in number or intensity but the epidermal frontal signals have increased in number and intensity. These signals are found in all larvae examined. In contrast, the subepidermal neuron-like signals observed earlier are not found in any larva at this developmental stage. Additional paired longitudinal signals are detected in the proboscis up to its posterior portion. Nerve plexus-like signals around the esophagus become evident (Figure 4D, ens). The majority of neuron-like signals is seen in the vicinity of the mouth opening. The neurite-like signals of the peripheral epidermal plexus are stronger, although neuron-like signals are not evident (Figure 4D, pep).

In larvae from 5 dpo onward, the neurite bundle-like signals of the brain and the lateral nerve cords become stronger and thus the associated neuron-like signals become relatively weaker (Figure 4D, bno). Therefore, only up to six neuron-like signals are counted in the frontal groups of lateral nerve cord neuron-like signals. A second pair of frontal neuron-like signals associated with the dorsal brain ring neurite-like signals is seen in some specimens. Thus, the number of neuron-like signals associated with the dorsal part of the brain ring amounts to four pairs. The signals remain weak throughout further development.

During subsequent development up to the oldest investigated larvae at 8 dpo, there are no new nervous system-like components observed. Most of the already formed neurite-like signals gain in intensity. As a consequence, the neuron-like signals become relatively weaker. Therefore, in the groups of neuron-like signals associated with the lateral nerve cord neurite-like signals five to six signals are seen in larvae at 6 dpo and in specimens at 8 dpo the number of clearly distinguishable signals amounts to four to five.

#### 3.1.4 Development of synapsin-like immunoreactivity

**FIGURE 5:**
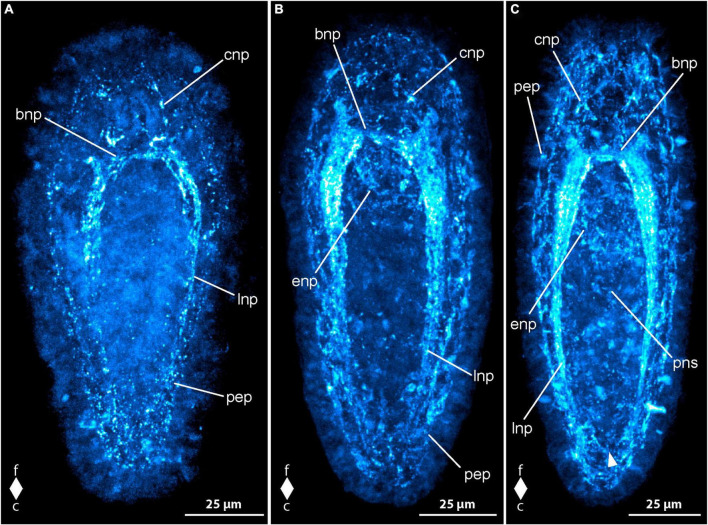
*Emplectonema gracile* larvae, synapsin-like immunoreactivity, maximum projection, frontal faces up in all images. **(A)** 2 days after oviposition, dorso-lateral view (*z* ≈ 42 μm, γ = 1.2); **(B)** 4 days after oviposition, dorsal view (*z* ≈ 51 μm, γ = 1.1); **(C)** 8 days after oviposition, ventral view (*z* ≈ 53 μm, γ = 0.8), note the caudal connection of the lateral longitudinal nerve cords by the anal commissure (arrowhead). bnp, brain ring neuropil; c, caudal; cnp, cephalic neuropil; enp, esophageal neuropil; f, frontal; lnp, longitudinal nerve cord neuropil; pep, peripheral epidermal plexus; pns, proboscis nervous system.

No synapsin-like immunoreactivity (syn-lir) is detected in any specimen before 2 dpo. At 2 dpo, all of the more advanced and already elongated larvae show neuropil-like signals in the brain ring (Figure 5A, bnp). The neuropil-like signals of the lateral nerve cords do not reach to the caudal end of the body and thus there is not caudal connection, the so-called anal commissure present (Figure 5A, lnp). Frontal of the brain ring signals, there are longitudinally oriented signals that might represent the neuropil-like component of the co-called cephalic nerves (Figure 5A, cnp). In some larvae an arcuate signal extends from one lateral side of the neuropil-like signal of the dorsal part of the brain ring to the other in a frontally convex way. This signal likely represents the syn-lir component of the basal neurites projecting from the subepidermal frontal 5HT-lir neuron-like signals to the dorso-lateral sides of the 5HT-lir brain ring neurite-like signals at this developmental stage. Inside the proboscis, a ring-shaped syn-lir signal is seen surrounding transition zone of the anterior and the posterior portion of the, at this developmental stage, bipartite proboscis. Many larvae also show clearly detectable signals of the peripheral epidermal plexus across the entire larval surface (Figure 5A, pep).

In larvae at 3 dpo, the brain ring neuropil-like signals have become stronger, especially in the ventral part. The signals of the cephalic nerves are clearly visible in all examined larvae, whereas the frontal bow-shaped signal has largely disappeared from most. The lateral nerve cord signals are extending up to the caudal end. A weak signal of the so-called anal commissure, represented by an arcuate dotted line of signals between the caudal ends of the lateral nerve cord signals is seen in many specimens. At this stage, additional weak neurite-like signals are detected in the anterior and middle part of the now tripartite proboscis. Additionally, paired neurite like signals extends from the lateral sides of the brain ring neuropil-like signals along the anterior up to the middle portion of the developing proboscis in some larvae. In most larvae, weak syn-lir signals are arranged around the esophagus up to the mouth opening. Syn-lir signals of the peripheral epidermal plexus are evident in most larvae at this developmental stage.

At 4 dpo, the brain ring neuropil-like signals are still stronger in the ventral than in the dorsal part (Figure 5B, bnp). Syn-lir signals of the cephalic nerves in the precerebral region are clearly seen in all larvae. The lateral nerve cord neuropil signals have become evident, especially in the anal commissure, that clearly shows in a larger fraction of investigated specimens. The same holds true for the signals of the plexus surrounding the esophagus and the signals of the peripheral epidermal plexus (Figure 5B, enp and pep). The proboscis neurite-like signals can be seen in the anterior and middle portion of the proboscis in most larvae.

In larvae at 5 dpo, all observed signals have increased in intensity, so that the anal commissure is clearly seen in nearly all specimens (Figure 5C, arrowhead). In the posterior portion of the proboscis, first weak syn-lir signals are visible (Figure 5C, pns). During further development, the syn-lir signals in the posterior portion of the proboscis become stronger, so that they are found in all larvae at 6 dpo. Between 6 dpo and the oldest stages investigated at 8 dpo, no further changes in the architecture of the syn-lir signals was observed.

### 3.2 *Cephalothrix rufifrons* (Archinemertea, Palaeonemertea)

#### 3.2.1 General development

The internally fertilized eggs of *C. rufifrons* are milky white and measure 80 μm in diameter. They are spawned enclosed in a thin-walled, translucent egg string. At 12°C, the early development until hatching takes about 3 days. A staging system like for monostiliferan species has never been published for palaeonemertean species, so that there are no specific names for the respective developmental stages. General landmarks to follow postembryonic development are the formation of the apical pit with the tuft of elongated cilia and the shifting of the mouth opening from the site of the blastopore to a fronto-ventral position. In *C. rufifrons* the appearance of a pair of dorso-lateral eyes, a caudally located tuft of elongated cilia, and the formation of the proboscis rudiment could be additionally documented (Figures 2C, D).

In freshly hatched larvae, gastrulation is complete 3 dpo, resulting in a spherical, completely ciliated body. Some larvae exhibit the beginning of the formation of an apical tuft of elongated cilia. By 4 dpo, larvae attain an ovoid body shape, with the ventrally shifted mouth opening located halfway along the body length. Both foregut and midgut are ciliated.

Asynchronous development, observed between 5 and 7 dpo, may be attributed to factors like internal fertilization or position of the egg in the egg string. Larvae display diverse developmental stages during this period. Some maintain an ovoid shape with a ventrally shifted mouth, while others elongate into a teardrop shape with the mouth positioned in the frontal half. A pair of shallow epidermal invaginations is evident on the lateral sides of the body anterior of the mouth opening in the majority of larvae. After 9 dpo, development becomes even more asynchronous, possibly due to competition for food. From this stage onward, food becomes crucial for further development. Larvae with a filled midgut have a spindle-shaped form, tapering more at the rear end (Figure 2D). Fed larvae increase significantly in size without altering their external shape. In the oldest stages examined, 42 dpo, the proboscis becomes recognizable as an epidermal invagination at the frontal end of the body (Figure 2E, pr).

#### 3.2.2 Development of FMRFamide immunoreactivity

**FIGURE 6:**
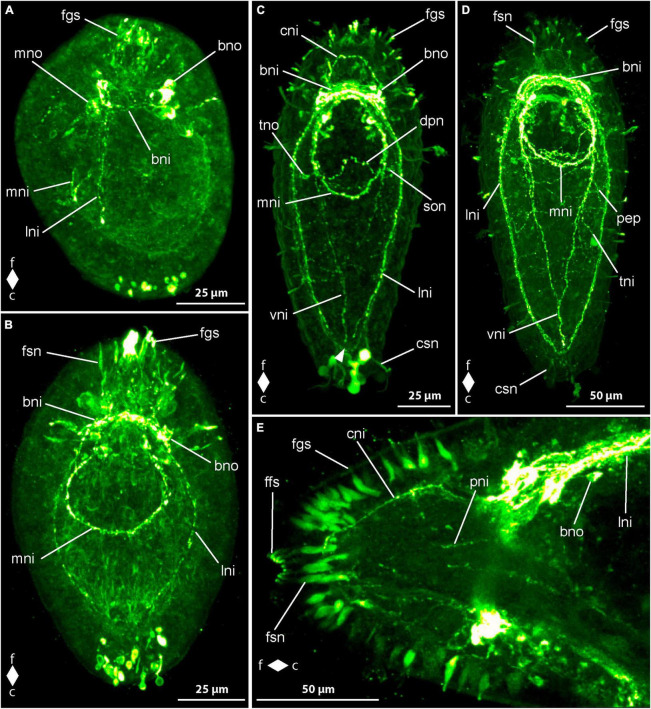
*Cephalothrix rufifrons* larvae, FMRFamide-like immunoreactivity, maximum projection, frontal faces up in panels **(A–D)** and left in panel **(E)**. **(A)** 4 days after oviposition, left lateral view, (*z* ≈ 72 μm, γ = 1.1); **(B)** 5 days after oviposition, ventro-lateral view (*z* ≈ 58 μm, γ = 1); **(C)** 14 days after oviposition, dorsal view (*z* ≈ 56 μm, γ = 0.8), note the caudal connection of the lateral longitudinal nerve cords by the anal commissure (arrowhead); **(D)** 21 days after oviposition, ventral view (*z* ≈ 79 μm, γ = 0.7); **(E)** 42 days after oviposition, ventro-lateral view (*z* ≈ 14 μm, γ = 0.8). bni, brain ring neurites; bno, brain ring neuron; c, caudal; cni, cephalic neurites; csn, caudal sensory neuron; dpn, dorsal pharyngeal neurite; f, frontal; ffs, frontal RFa-lir signals; fgs, frontal gland/sensory cell signals; fsn, frontal sensory neuron; lni, longitudinal nerve cord neurites; mni, mouth ring neurites; mno, mouth ring neuron; pep, peripheral epidermal plexus; pni, proboscis neurites; son, suboral neurite; tni, transversal lateral neurite; tno, transversal lateral neuron; vni, ventral neurite.

In newly hatched larvae, observed 3 dpo, only a few weak and short neurite-like RFa-lir signals are evident along the subapical region between the animal pole of the gastrula and the cells of the primordial gut. Further similar signals can be detected along the sides of the primordial gut in some larvae. By 4 dpo, the larvae’s body elongates, and the first RFa-lir signal of the neurites of the brain ring appears. This signal extends in a ring shape frontally of the intestine, transverse to the longitudinal axis of the body and on the dorso-lateral sides of the body. Additionally, a pair of spherical neuron-like signals, connected to the brain ring via short neurite-like signals, becomes visible (Figure 6A, bno). Caudal to the brain ring, the first neurite-like signals of the longitudinal nerve cords are weakly visible in some larvae, reaching backward up to a maximum of half the body length. Paired signals of the neurites of the mouth ring are recognized lateral to the mouth opening, although no connection between them behind the mouth opening is observed at this developmental stage. In the frontal sections of the oral neurite-like signals, near the signals of the ventral part of the brain ring neurite, the oral neurite-like signals exhibit a pair of spherical neuron-like signals on each side of the mouth opening (Figure 6A, mno). At the frontal end of the larva, around the apical pit where the apical cilium inserts, up to five narrow, spindle-shaped RFa-lir signals are observed. These signals could potentially be apical sensory neurons or gland cells whose secretion has an affinity for the antibodies or fluorescent dyes used. At the posterior end, mucous gland secretions are also clearly visible, some of which are still present in the gland cells, and some have leaked out, showing an affinity for the secondary antibody used or for its conjugated fluorescent dye (Figure 6A, fgs). The cilia of the apical cilium are also labeled in some larvae, presumably indicating glandular secretions with an affinity to one of the antibodies used.

Between 5 and 7 dpo, the larvae elongate slightly, showing up to three pairs of neuron-like signals on the dorsal side of the neurite-like signal of the brain ring. These signals are arranged in groups on both sides of the midline and are connected to the dorso-median section of the brain ring neurite-like signal via another neurite-like signal. Most signals appear caudal to the brain ring, with a maximum of one pair found frontal to the brain ring signal. The neurite-like signals of the longitudinal nerve cords extend caudally up to two-thirds to about four-fifths of the body length in more developed larvae (Figure 6B, lni). A caudal connection of the neurite-like signals of the longitudinal nerve cords, known as the anal commissure, is not formed in any examined larvae at this stage. The neurite-like signals of the oral ring neurite bundle also extend caudally, connected by a weak, arcuate neurite-like signal on the caudal side of the mouth opening in some more developed larvae (Figure 6B, mni). The number of neuron-like signals of the mouth ring increases to two pairs in more developed larvae, all found in the fronto-lateral section of the mouth ring neurite-like signal. The status of closure of the frontal section of the oral ring neurite-like signal is unclear, as it is directly adjacent to the ventral section of the brain ring neurite-like signal. The epidermal, spindle-shaped signals, possibly representing sensory or gland cells, are more numerous and occur not only terminally but also subterminally at the frontal body pole.

After 9 dpo, the number of neuron-like signals associated with the dorsal section of the neurite-like signal of the brain ring has increased to up to six pairs. A signal extending in a horizontal arc into the frontal end of the body is observed in front of the brain, originating from both lateral sides of the brain ring neurite-like signal. This signal most likely corresponds to the so-called cephalic nerves (Figure 6C, cni). The neurite-like signals of the longitudinal nerve cords extend to the posterior end of the larva and appear to be composed of more than one neurite-like signal per body side. However, an anal commissure is only clearly recognizable in about half of the larvae examined (Figure 6C, arrowhead). The signals of the oral ring neurite bundle are wider in slightly more than half of the larvae and seem to be composed of more than one signal. In many larvae, the frontal section of the oral ring neurite bundle connects to the ventral signals of the brain ring neurites near the transition to the longitudinal nerve cords via a pair of weak, fronto-lateral neurite-like signals, referred to as preoral neurites. In the posterior third of the oral ring neurite bundle, a neurite-like signal runs in a dorsal arc from the lateral sides of the oral ring neurite-like signals, termed herein the dorsal pharyngeal nerve (Figure 6C, dpn). In about two-thirds of the larvae, the neurite-like signal extends to both lateral sides and runs as so-called suboral neurites in a wide, frontally open arc to the dorso-lateral sides of the body. It crosses the signals of the longitudinal nerve cords and ends in some larvae in an ovoid neuron-like signal, termed transversal lateral neuron (Figure 6C, tno). A pair of similar neurite-like signals, originating at the level of the longitudinal nerve cords and running dorso-laterally, can be detected in the posterior third of the body in about half of the larvae examined. On the ventral side, the neurite-like signals of the so-called ventral nerve can be detected in some larvae. It consists of two roots that run diagonally from each side from the caudal edge of the oral ring neurite-like signal to the ventral midline and connect with each other about halfway along their course to the posterior end (Figure 6C, vni). At the rear end of the larvae, weak epidermal signals can be detected, which, with their narrow, spindle-shaped form, resemble the signals in the epidermis at the front end of the larva and could therefore also be interpreted as signals from sensory cells or gland cells.

In larvae at 14 dpo, the brain ring signal on the ventral side consists of up to four parallel strands, with the most caudal likely corresponding to the frontal arch of the oral ring neurite bundle. On the dorsal side of the body, up to two neurite bundle signals can be recognized in the brain ring. The number of neuron-like signals associated with the dorsal brain ring neurite-like signal, located dorso-lateral to the dorsal midline, is not noticeably increased compared to the earlier developmental stage examined. Small, frontally tapered pyriform neuron-like signals are evident in some larvae, mainly occurring in dorso-median positions. The anal commissure, connecting the posterior ends of the neurite-like signals of the longitudinal nerve cords in an arcuate shape, is clearly detectable in all larvae, and the dorsal pharyngeal nerve and the ventral nerve are also found in all larvae examined (Figure 6D). Of the neurite-like signals of the oral ring, the preoral neurite-like signals are more clearly visible in many more larvae than before, and the oral ring neurite bundle clearly shows several neurite-like signals. One to two pairs of weak, narrow, elongated signals can be detected on each side of the mouth opening within the oral ring signal. It cannot be conclusively determined whether these signals are sensory cells or glandular cells.

After 21 dpo, a few larvae also show a pair of ovoid, bilaterally arranged neuron-like signals on the ventral side of the brain ring signal. On the dorsal side, numerous neuron-like signals can be recognized, mainly located caudal to the neurite-like signals of the brain ring. The horizontal, arcuate signal of the precerebral neurites frontal to the brain ring ligament is visible in all larvae and has two separate roots to the brain ring signal. The neurite-like signals of the ventral nerve and the suboral neurites are clearly visible in all larvae examined, whereas similar neurite-like signals, termed transversal neurites, which are located further back and originate from the neurite-like signals of the longitudinal nerves, are only clearly visible in slightly less than half of the larvae. The paired signals on both sides of the mouth opening, interpreted as sensory or glandular cells, have become more intense in most larvae at this stage of development. Weak, reticularly arranged neurite-like signals, possibly attributable to the peripheral epidermal plexus, occur mainly on the dorsal side of the body of the larvae examined (Figure 6D, pep).

In the larvae after 28 days of development, three neurite bundle signals can be clearly distinguished in the ventral section of the brain ring. On the dorsal side, the brain ring is composed of two neurite bundle signals. In many larvae, a pair of neuron-like signals is associated with the lateral sides of the ventral section of the brain ring neurite bundles. The number of neuron-like signals located caudally posterior to the dorsal portion of the brain ring has further increased, and a pair of narrow elongated neuron-like signals, located on either side of the dorsal midline and aligned parallel to the longitudinal body axis, is clearly visible anterior to the dorsal brain ring signal in the majority of larvae. On each side of the mouth ring neurite-like signal, up to two neuron-like signals can be recognized fronto-laterally. At the level of the posterior edge of the frontal third of the neurite-like signals of the longitudinal nerve cords, there is also a group of up to three ovoid neuron-like signals on each side of the body.

In the oldest stages examined, 42 days after egg laying, the signals of the brain ring and the longitudinal nerve cords are clearly more prominent than in previous developmental stages. As a result, the other signals of the nervous system appear relatively weaker. The signals of the longitudinal nerve cords are broader, show several neurite-like signals, and clearly show sub-compartments (Figure 6E). In about half of the larvae examined, the oral ring neurite bundle has two additional paired neuron-like signals associated with it, each of which is located slightly more caudally, about halfway along the oral ring neurite bundle on each side of the body. In the most developed larvae of this developmental stage, a clear spatial separation of the frontal section of the oral ring signal from the ventral section of the brain ring signal can be observed. The apices of the cells that form the apical pit uniformly show frontal RFa-lir signals that might be continued more basally in the cells as frontal sensory neuron-like signals (Figure 6E, ffs). A pair of longitudinal neurite-like signals extends longitudinally along the developing proboscis (Figure 6E, pni).

#### 3.2.3 Development of serotonin immunoreactivity

In newly hatched larvae, observed 3 dpo, a few dot-shaped signals arranged in short, curved lines are visible around the primordial gut and near the anterior pole of the gastrula. No pronounced neurite or neuron-like signals are observed.

**FIGURE 7:**
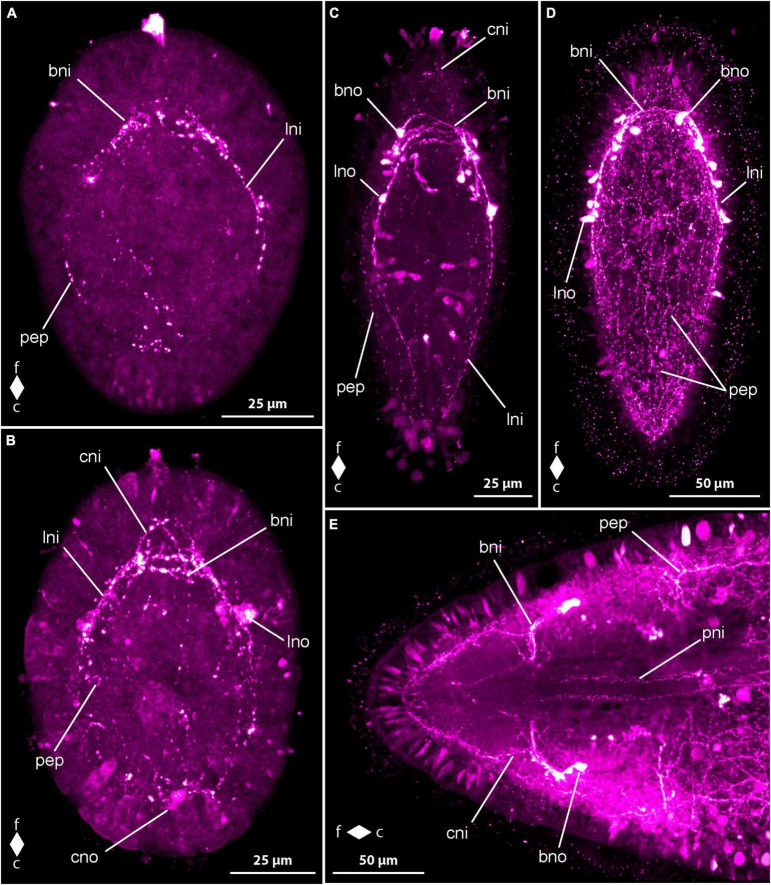
*Cephalothrix rufifrons* larvae, serotonin-like immunoreactivity, maximum projection, frontal faces up in panels **(A–D)** and left in panel **(E)**. **(A)** 4 days after oviposition, ventral view (*z* ≈ 73 μm, γ = 1.2); **(B)** 7 days after oviposition, dorsal view (*z* ≈ 56 μm, γ = 0.8); **(C)** 9 days after oviposition, dorsal view (*z* ≈ 63 μm, γ = 0.8); **(D)** 21 days after oviposition, dorsal view (*z* ≈ 84 μm, γ = 1.2); **(E)** 42 days after oviposition, ventral view (*z* ≈ 14 μm, γ = 0.8). bni, brain ring neurites; bno, brain ring neuron; c, caudal; cni, cephalic neurites; f, frontal; lni, longitudinal nerve cord neurites; cno, caudal neuron; lno, longitudinal nerve cord neuron; pep, peripheral epidermal plexus; pni, proboscis neurites.

By 4 dpo, one or a few clear signals can be detected in the area below the apical ciliated pit, extending outside the body along the cilia. These signals likely represent glandular secretions rather than neurons, displaying an affinity for the fluorescent dye of the secondary antibody (Figure 7A, arrowhead). Punctate 5HT-lir signals, arranged in lines, are more concentrated in the frontal part of the body, exhibiting a ring-shaped arrangement perpendicular to the longitudinal axis. These likely represent the initial 5HT-lir neurite-like signals of the brain ring. In the caudal section of the neurite-like signal ring, the dot-like lines mostly run parallel to the longitudinal axis, possibly indicating the first neurite-like signals of the lateral longitudinal nerve cords (Figure 7A, lni). Punctate signals in the caudal half of the larva early might represent signals of a peripheral epidermal nerve plexus (Figure 7A, pep).

Most larvae between 5 and 7 dpo exhibit recognizable neurite-like signals of the dorsal and ventral sections of the brain ring, mainly in the form of dot-like signals arranged in lines (Figure 7B, bni). In the caudal section of the larvae, similar punctate signals are irregularly distributed, suggesting they are mainly the 5HT-lir signals of the epidermal nerve plexus (Figure 7B, pep). Neuron-like signals caudal and frontal to the mouth opening, especially on the dorso-lateral sides of the body, are often present, sometimes arranged in pairs, not obviously associated with the neurite-like signals of the longitudinal nerve cords or the brain ring, but close to signals that could be assigned to the epidermal neurite plexus. The number of these signals does not seem to correlate with the developmental progress of the larvae. In some, especially less developed larvae, a few cells in the epidermis exhibit a brighter, diffuse signal than other epidermal cells. Presumably, these lighter epidermal cells represent early differentiation stages that later become neurons of the peripheral epidermal plexus by sinking below the epidermis. Due to the few neuron-like signals in the peripheral epidermal plexus, it can be assumed that the perikarya of these neurons no longer show a pronounced 5HT-lir signal in the further course of their differentiation. Signals associated with the brain ring, possibly corresponding to the signals of the longitudinal nerve cords, extending up to the caudal edge of the mouth opening, are observed in more developed larvae. Other punctate signals associated with the brain ring and arranged in lines likely represent neurites of the cephalic nerves or the precerebral component of the peripheral epidermal plexus. Paired neuron-like signals can be detected on each side frontal to the mouth opening, connecting with the longitudinal 5HT-lir neurite-like signal of the longitudinal nerve cord on that side. The less developed larvae often exhibit a corresponding neuron-like signal on one side only (Figure 7B, lno). In more developed larvae, a dorso-median, ovoid, subterminal neuron-like signal is observed at the rear end of the larva, occasionally accompanied by a second, slightly more frontally located neuron-like signal (Figure 7B, cno).

At 9 dpo, at least one continuous, ring-shaped neurite-like signal can be recognized in the brain ring in both the dorsal and ventral sections, completing the closure of the brain ring. In some larvae, the ventral section of the brain ring exhibits two neurite-like signals. Paired groups of up to two neuron-like signals are connected to the dorsal section of the brain ring neurite-like signal via a short neurite-like signal on each side of the midline (Figure 7C, bno). Another unpaired neuron-like signal on one side of the body is occasionally part of the group of dorsal neuron-like signals in some larvae. Two paired neuron-like signals can be observed on the ventral side of the brain ring, each located ventro-laterally. Occasionally, in a few larvae, an unpaired, weaker neuron-like signal can be detected on one side of the ventral section of the brain ring. The paired neurite-like signals of the longitudinal nerve cords on each side of the body are almost completely continuous and extend almost to the caudal end of the larva (Figure 7C, lni). A so-called anal commissure, connecting the neurite-like signals of the longitudinal nerve cords at the posterior end, cannot be reliably detected. Along the signals of the longitudinal nerve cords, a neuron-like signal can be observed on each side of the body approximately at the posterior edge of the anterior third of the body length (Figure 7C, lno). In a few larvae, an additional, unpaired signal can be seen near one of the paired signals on one side of the body. A caudal neuron-like signal, slightly subterminal and median on the dorsal side of the larva in younger developmental stages, is only found in a few larvae. A pair of mostly elongated, spindle-shaped signals, which show clear differences in shape in different larvae, is located on each side at the fronto-lateral edge of the mouth opening. Additionally, in some larvae, a single, similar but weaker signal can be seen on the frontal edge of the mouth opening. It is not clear whether these signals are signals from sensory cells or glandular secretions that have an unspecific affinity for the antibodies or fluorescent dyes used. Neurite-like signals of the peripheral epidermal plexus can be recognized on all sides along the length of the body both frontally and caudally of the brain ring signals (Figure 7C, pep). Additional unpaired neuron-like signals in dorso-lateral and ventro-lateral positions caudal to the brain ring are not found in all larvae and not at the same location. It can be assumed that even at this stage of development, early neurons belonging to the peripheral epidermal plexus are still present and show only a temporarily limited perikarya-like signal.

In the further development of the 5HT-lir nervous system up to 28 dpo, no new components of the nervous system appear. Instead, those already formed are further elaborated. This is especially true for the peripheral epidermal plexus neurite-like signals (Figure 7D, pep). The body length of the larvae increases successively, whereby the body section in front of the brain appears to lengthen somewhat more in relative terms. The number of parallel neurite-like signals in the ventral section of the brain ring neuropil is four in 21–28 dpo larvae and five in the oldest stages examined after 42 days (Figure 7D, bni). The neurite-like signal of the dorsal brain ring neuropil shows a splitting into two parallel signals running transverse to the longitudinal axis of the body after 21 days of development, whereby a weak signal of splitting can also be seen in individual specimens in earlier stages. In one of the 42-day-old larvae examined, a weak third neurite-like signal running parallel to the other two was detected. A neurite-like signal of the so-called anal commissure, which connects the signals of the longitudinal nerve cords at their caudal end, is already weakly visible after 11 days and clearly visible in all examined larvae after 21 days of development.

The number of neuron-like signals associated with the neurite-like signals of the longitudinal nerve cords only increases after 11 dpo of development and amounts to between two and four paired signals on the sides of the body in the larvae examined after 21 dpo (Figure 7D, lno). In the larvae, in which four pairs occur, these are arranged in two groups of two signals each, one of which is located on the caudal edge of the anterior third of the body, the other further caudally, approximately halfway along the body. In the further course of development, the number of neuron-like signals associated with the lateral nerve cords increases further, so that numerous signals can be detected in larvae at 28–42 dpo, which no longer appear to be arranged strictly in pairs on both sides of the body. The number of neuron-like signals associated with the ventral section of the brain ring neurite-like signals increases to two pairs in 21 dpo larvae and remains so until the oldest stages examined at 42 dpo. On the dorsal side of the brain ring neuropil-like signals, the number of neuron-like signals per group increases to three in some larvae at 21 dpo. At later developmental stages (28–42 dpo), three neuron-like signals on each side are associated with the dorso-lateral section of the brain ring neuropil in almost all larvae examined. The neurite-like signals of the peripheral epidermal plexus become more intense as development progresses and appear to form a denser network. A pair of faint neurite plexus-like signals extends longitudinally along the developing proboscis at 42 dpo (Figure 7E, pni). Up to this stage of development, there are no signs of 5HT-lir signals from the nervous system in the developing foregut or midgut.

#### 3.2.4 Development of synapsin immunoreactivity

**FIGURE 8:**
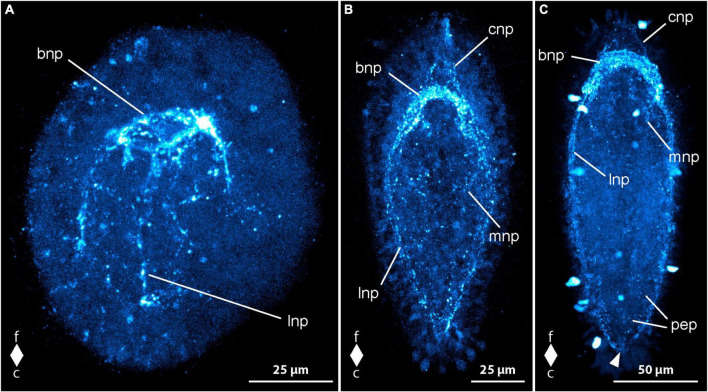
*Cephalothrix rufifrons* larvae, synapsin-like immunoreactivity, maximum projection, frontal faces up in all images. **(A)** 4 days after oviposition, dorso-lateral view (*z* ≈ 72 μm, γ = 0.9); **(B)** 9 days after oviposition, dorsal view (*z* ≈ 58 μm, γ = 1.1); **(C)** 21 days after oviposition, ventral view (*z* ≈ 62 μm, γ = 1.3), note the caudal connection of the lateral longitudinal nerve cords by the anal commissure (arrowhead). bnp, brain ring neuropil; c, caudal; cnp, cephalic neuropil; f, frontal; lnp, longitudinal nerve cord neuropil; mnp, mouth ring neuropil; pep, peripheral epidermal plexus.

The first syn-lir signals, attributable to nervous system components, become evident in *C. rufifrons* larvae 4 dpo. At 3 dpo, newly hatched gastrula larvae display sparse punctiform signals arranged dorsolaterally along the primitive gut. By 4 dpo, distinct signals form a closed ring near the frontal end, likely representing the developing neuropil of the brain ring (Figure 8A, bnp). Some larvae exhibit weaker signals extending ventro-laterally, possibly indicating paired signals of the longitudinal nerve cords (Figure 8A, lnp) and, ventrally, early signals of the fronto-lateral neurite bundle ring around the mouth opening. Further along the body, irregularly arranged punctiform signals, presumed to be part of the peripheral epidermal plexus, are observed. These signals are more concentrated caudally to the brain ring signal.

Peripheral syn-lir signals manifest in all larvae 5 dpo, and some larvae show intensified signals in the lateral sections of the oral ring neurite bundle (Figure 8B, mnp). At 7 dpo, longitudinal nerve cord signals extend caudally, but reach the caudal half of the larva only as dot-shaped signals. The anal commissure, connecting the longitudinal nerve cords caudally, is only faintly developed. Additional peripheral syn-lir signals, likely demarcating developing cephalic neuropil, appear frontal to the brain ring.

In 9 dpo-larvae, the peripheral frontal signals form arcuate lines along the pre-cerebral body section, indicating the cephalic neuropil (Figure 8B, cnp). The brain ring neuropil-like signal strengthens, with ventral and lateral sections exhibiting broader signals (Figure 8B, bnp). The dorsal section remains thin. Between 11 and 14 dpo, longitudinal nerve cord signals extend to the caudal end, connecting through a continuous anal commissure (Figure 8C, arrowhead). The caudal arch of the oral ring neurite bundle becomes more noticeable. In 21 dpo-larvae, syn-lir signals are evident in the brain ring, longitudinal nerve cords, anal commissure, lateral sections of the oral ring, and the peripheral epidermal plexus (Figure 8C, pep). The dorsal section of the brain ring neuropil-like signal broadens. Up to 42 dpo, no significant changes beyond general growth occur in the nervous system components. In advanced stages, the peripheral epidermal plexus forms distinct net-like lines, and syn-lir signals extend frontally from the dorso-lateral and ventro-lateral faces of the brain ring. The caudal arch of the oral ring neurite bundle remains weak. The broad ventral and ventro-lateral sections of the brain ring signal contrast with the still thinner dorsal section. No syn-lir nervous system-like signals are detected in the developing proboscis.

## 4 Discussion

Generally, RFa-lir is not completely unproblematic in that neuron-like signals are often not consistently showing. Thus, their recorded numbers are prone to considerable variation, even between members of the same developmental stage. Serotonin-like immunoreactivity (5HT-lir), especially in neuron-like signals, shows more consistency between larvae of the same age compared to RFa-lir. Interestingly, neuron-like signals, especially of the signals associated with the neurite-like signals of the lateral nerve cords becomes inconspicuous in older stages, so that reporting exact numbers in these is not always possible. Serotonin-antibodies of two different manufacturers were used in different batches of larvae of *E. gracile*, but the two different antibodies did not yield different staining results.

Using antibodies against acetylated α-tubulin in nemertean larvae, as it has become a standard in many studies on neurodevelopment of larvae, comes with major impediments to visualization of the signals. Employing any tubulin antibody leads to staining of the tubulin component not only of neurites but also of cilia. The staining of cilia is of special concern in nemertean larvae since they are completely covered with densely set cilia that outshine the tubulin-lir signals in the neurites. Thus, maximum projections of complete specimens, as mostly used herein, will only show the external ciliation. Projections of only the middle layers on the other hand, will only show dimly the more prominent components of the developing nervous system, such as brain and lateral nerve cords, which in this investigation more distinctly labeled with antibodies against synapsin.

Currently, data on the development of the nervous system by means of immunohistochemistry against serotonin and FMRF-amide combined with analysis by confocal laser scanning microscopy are available for a handful of species from all three high-ranking clades of Nemertea (Hay-Schmidt, 1990; Chernyshev and Magarlamov, 2010; Maslakova, 2010a; Hindinger et al., 2013; Hiebert and Maslakova, 2015; von Döhren, 2016b,^2021^). However, both antibodies have so far only been studied in two species, *Lineus albocinctus* (Heteronemertea) (Hindinger et al., 2013) and *Carinina ochracea* (Carinomiformes) (von Döhren, 2016b). For development of immunoreactivity against synapsin, there are only data available on the paleonemertean species *C. ochracea* (von Döhren, 2016b). Thus, this article provides the first report on the development of the syn-lir component of the nervous system in a hoplonemertean species.

### 4.1 The components of the nervous system are largely similar in larvae of palaeonemertean and hoplonemertean species

**FIGURE 9:**
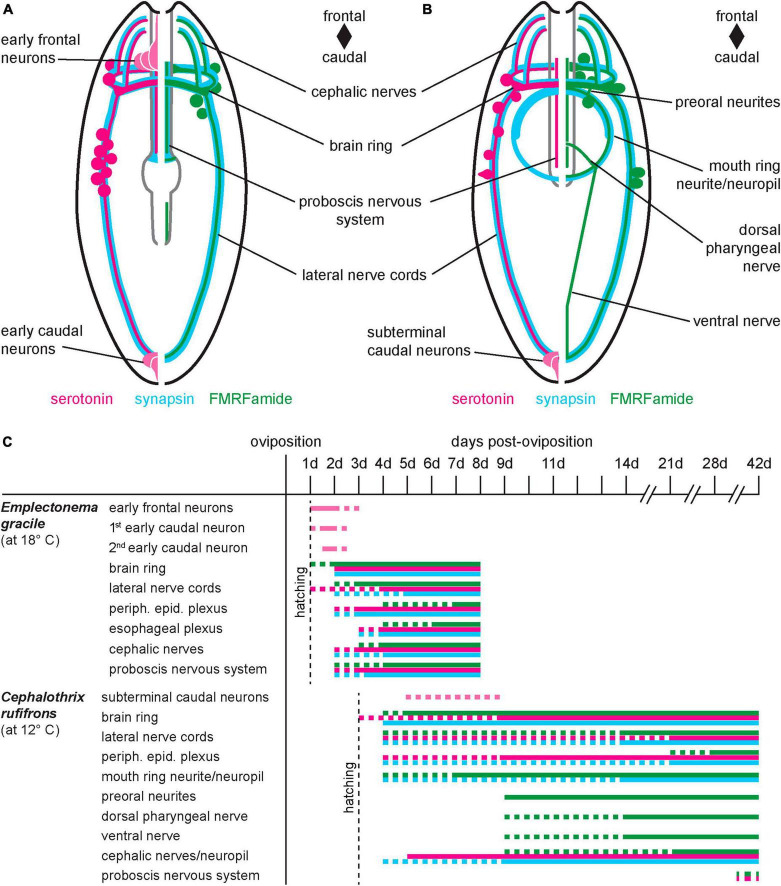
Comparative summary of nervous system development. **(A)** *Emplectonema gracile*, ventral view, schematic representation of nervous system in 8 dpo larvae; **(B)** *Cephalothrix rufifrons*, ventral view, schematic representation of nervous system in 42 dpo larvae; larvae are not shown to scale, esophageal plexus in *E. gracile*, peripheral sensory cells and peripheral epidermal plexus in both species omitted for clarity; **(C)** schematic timeline of development of major components of the nervous system (color coding as in panels **A,B**, vertical dotted line indicates time of hatching, horizontal dotted lines indicate not completely formed or degenerating structures).

5HT-lir and RFa-lir is seen in most of the components of the developing nervous system in *E. gracile* and *C. rufifrons*, namely the brain ring, the longitudinal nerve cords, the peripheral epidermal plexus, the cephalic neurites, and the nervous system of the proboscis (Figures 9A,, peripheral epidermal plexus not shown). In *C. ochracea*, RFa-lir in the peripheral epidermal plexus has reported to be absent (von Döhren, 2016b). This absence is likely attributable to the oldest larval stage of this species being less far developed than the larval stages of *C. rufifrons*, the paleonemertean species investigated in the present study. Several accounts on the architecture and immunoreactivity against serotonin and FMRFamide antibodies of the adult nervous system in paleo- and hoplonemertean species support the hypothesis that the components seen in the larvae represent structures of the future adult nervous system (Figure 9C; Punin et al., 2003; Zaitseva et al., 2007; Beckers et al., 2013, 2018; Zaitseva and Petrov, 2013). Interestingly, in contrast to the developing proboscis in *C. rufifrons*, there seems to be no 5HT-lir in the longitudinal proboscis neurites in adults of the closely related palaeonemertean species *Procephalothrix filiformis* (Archinemertea) and *Cephalothrix linearis* (Archinemertea) (Beckers et al., 2013; Zaitseva and Petrov, 2013). Whether the difference in immunoreactivity of nervous system components in cephalothricid Palaeonemertea represents interspecific differences or is due to the 5HT-lir in the proboscis nerves being restricted to a certain developmental period needs additional comparative investigations to be decided. In the developing juvenile of Pilidiophora inside the pilidial envelope, the developing proboscis also displays 5HT-lir neurite processes (Hay-Schmidt, 1990; Maslakova, 2010a). A dorsal nerve that is present in the larvae of *C. ochracea* and *Tubulanus polymorphus* (Tubulaniformes), is absent from the larva of *C. rufifrons*, although it is present in adults (Beckers et al., 2013; von Döhren, 2016b,^2021^). In *Carinoma* species (Carinomiformes), the dorsal nerve is also present in adults but has not yet formed in the investigated larval stages (Beckers et al., 2013; von Döhren, 2021).

In *C. rufifrons*, the components of the stomatogastric nervous system, i.e., the mouth ring neurite bundle, preoral neurite bundles, and the suboral neurites that fuse to the so-called ventral nerve in *C. rufifrons* are devoid of 5HT-lir (Figure 9B). The same is reported in the paleonemertean species *C. ochracea* (von Döhren, 2016b). Absence of 5HT-lir elements in the stomatogastric nervous system seems to be attributable to the developmental stage, since 5HT-lir elements are readily found in the adult gastrodermal plexus of species of Palaeonemertea (Markosova et al., 2007; Zaitseva and Petrov, 2013: *C. linearis*), Heteronemertea (Punin et al., 2003; Markosova et al., 2007; Beckers et al., 2011: *Lineus ruber*, *Lineus viridis*; Zaitseva and Petrov, 2013: *L. ruber*), and Monostilifera (Markosova et al., 2007; Beckers et al., 2018: *Amphiporus lactifloreus*; Zaitseva and Petrov, 2013: *Tetrastemma* cf. *candidum*). A serotonergic stomatogastric subset of the nervous system comprising an oral neurite ring and paired suboral neurites is present already in the early pilidium larva of *L. albocinctus* (Hay-Schmidt, 1990; Hindinger et al., 2013). In this species, there seem to be no RFa-lir components in the stomatogastric nervous system of the young larval stages (Hay-Schmidt, 1990; Hindinger et al., 2013). These observed differences in immunoreactivity of the stomatogastric subset of the nervous system between the larvae of Heteronemertea on the one hand and Monostilifera and Palaeonemertea on the other hand lend additional support to the hypothesis of the derived nature of the pilidium larva within Nemertea.

Syn-lir is also abundant in the developing nervous system of Nemertea, namely in the brain ring, the longitudinal nerve cords, the peripheral epidermal plexus, the cephalic neurites and large parts of the stomatogastric nervous system in both species documented in this study (Figures 9A, B, esophageal plexus in *E. gracile* and peripheral epidermal plexus in both species not shown). This inventory of immunoreactivity is largely similar to that reported in developing larvae of *C. ochracea*, the only other species that detailed, comprehensive data is available for. In *C. ochracea* however, cephalic neurites show no evident RFa- or syn-lir (von Döhren, 2016b). In *E. gracile*, elements of the nervous system in the proboscis also show syn-lir (Figure 9A). In adult Nemertea, syn-lir has so far only been studied in the neuropil of the longitudinal nerve cords of the pilidiophoran species *L. viridis* (Beckers, 2015) but it is expected to be present in other species and nervous system components as well. The data strongly speak for a medullary cord-type organization of the main components of the nervous system not only in the adult but also throughout development.

### 4.2 The sequence of nervous system formation in Palaeonemertea and Hoplonemertea shows only minor differences that are congruent with general developmental characteristics

Development of the respective nervous system components in *C. rufifrons* and *E. gracile* is largely similar to their relatives within Palaeonemertea and Hoplonemertea, respectively. Generally, the main components, i.e., the ring-shaped brain and the paired longitudinal nerve cords, develop in a frontal to caudal progression in all investigated paleonemertean and hoplonemertean species (Figure 9C; Chernyshev and Magarlamov, 2010; Hiebert and Maslakova, 2015; von Döhren, 2016b,^2021^). Whereas the 5HT-lir signals in the peripheral epidermal plexus of *C. rufifrons* and *E. gracile* are seen almost as early as the onset of brain development, the RFa-lir signals in this part of the nervous system are seen much later, after the brain and the longitudinal nerve cords have already progressed considerably in their development (Figure 9C).

Of considerable interest are the 5HT-lir elements of the nervous system that develop before the onset of development of brain and peripheral epidermal plexus. In *E. gracile*, several frontal neuron-like signals, some with thin processes to the apical surface and a single caudal neuron-like signal with an apical process are visible in the youngest larvae investigated. Shortly after, a second caudal neuron is present. Similar neurons, termed apical neurons and additional apical neurons at the frontal end and a caudal neuron at the caudal end, are visible in *Pantinonemertes californiensis* (Monostilifera) and *Quasitetrastemma stimpsoni* (Monostilifera) in comparable stages of development, although the number of frontal signals differs. An additional pair of so-called subapical signals is present in *P. californiensis* and *Q. stimpsoni* that seems to be absent from *E. gracile*. As in *E. gracile*, most, if not all of these early neurons either lose their 5HT-lir or degenerate altogether during the course of further development (Figure 9C; Chernyshev and Magarlamov, 2010; Hiebert and Maslakova, 2015). The apical neurons and most likely also the additional apical neurons are homologous to the frontal neurons of *E. gracile*. The caudal neuron seen in the other hoplonemertean species is homologous to one of the caudal neurons in *E. gracile*, most likely the one that appears first. In larvae of *C. rufifrons*, there is no indication of frontal transitory 5HT-lir neurons (Figure 9B). However, in the other investigated palaeonemertean species, *C. ochracea*, the first 5HT-lir signals comprise two frontal neuron-like signals with apical processes and two caudal neuron-like signals, all of which possessing an apical process to the larval surface (von Döhren, 2016b). In the light of current hypotheses on phylogeny of Nemertea that state monophyly of Palaeonemertea (Andrade et al., 2014; Kvist et al., 2014, 2015), it is most parsimonious to assume the early, transitory 5HT-lir frontal neuron-like signals have been reduced in *C. rufifrons*. From the data at hand, it does not become clear however, whether the neurons are there and only 5HT-lir is absent or whether the neurons are completely reduced. One or two transitorily expressed caudal neuron-like signals are seen in some larvae of *C. rufifrons* after the appearance of 5HT-lir in the brain ring and longitudinal nerve cords (Figure 9C). It is possible that these caudal neurons are homologous to the early caudal 5HT-lir signals observed in *C. ochracea* and the investigated hoplonemertean species but their expression has been heterochronically shifted to a later time point in development in *C. rufifrons*. Transitory RFa-lir is present in form of one or two faint, frontal neuron-like signals with an apical process in *T. polymorphus* and *Carinoma* species (von Döhren, 2021). Whether these faint signals are also present in larvae of other nemertean species and have only been overlooked due to their faintness and short-lived nature, presently remains unclear.

In all palaeonemertean species studied so far, immunoreactivity against serotonin, FMRFamide, and synapsin is not detectable until after hatching (Figure 9C; von Döhren, 2016b,^2021^). In all investigated hoplonemertean species, however, the timing of the development of the nervous system components relative to hatching is different (Chernyshev and Magarlamov, 2010; Hiebert and Maslakova, 2015; von Döhren, 2021). Whereas larvae of *Carcinonemertes carcinophila* (Monostilifera) and *Amphiporus* sp. (Monostilifera) hatch as developmental stages that have already formed the brain ring and have begun to form the longitudinal nerve cords, the newly hatched larva of *E. gracile* does not show any sign of these nervous system components (Figure 9C; von Döhren, 2021). In *Q. stimpsoni* and *P. californiensis*, and in the hoplonemertean species *Oerstedia dorsalis* (Monostilifera), the larva hatches at a similarly early developmental stage (termed invagination stage in Hiebert et al., 2010) as the larva of *E. gracile* (Chernyshev, 2008; Maslakova and von Döhren, 2009; Chernyshev and Magarlamov, 2010; Hiebert and Maslakova, 2015). In the light of current phylogenetic hypotheses of Hoplonemertea (Kvist et al., 2014, 2015), it is most parsimonious to assume that formation of the nervous system after hatching is the ancestral state in the development of hoplonemertean species. Moreover, since formation of the nervous system after hatching is also a characteristic of the development of palaeonemertean larvae (Figure 9C; von Döhren, 2016b,^2021^), it is very likely an ancestral trait of development in all of Nemertea.

The proboscis apparatus in all investigated hoplonemertean species, and hence its nervous system develops earlier than in the investigated paleonemertean species (Iwata, 1960; Chernyshev and Magarlamov, 2010; Hiebert and Maslakova, 2015; von Döhren, 2016b,^2021^). This is also the case in *E. gracile* as opposed to the palaeonemertean species *C. rufifrons*, in which the developing proboscis nervous system is detectable only after 42 days of development (Figure 9C). Thus, this study is the first to provide data on the development of the nervous system of the proboscis apparatus in Palaeonemertea. The larvae of the monostiliferan species *Emplectonema viride* have been shown to use their proboscis for prey capture early on in their life (Mendes et al., 2023). The data for *C. rufifrons* suggest that the proboscis is most likely not yet functional up to 42 dpo and maybe even until settlement and therefore cannot be used to capture prey. Therefore, although the larvae have been shown to ingest larger food items it is not yet clear how these are captured without a proboscis. Taking into account that the proboscis in pilidium larvae of Heteronemertea also develops relatively late (Maslakova, 2010a), it is more parsimonious to assume that an early formed proboscis is a derived characteristic in Nemertea which is restricted to Hoplonemertea.

### 4.3 Nervous system development in Nemertea shows several traits that are most likely ancestral for spiralian nervous system development

The derived nature of the pilidium larva precludes this larval type from comparisons with the larval types observed in paleonemertean and hoplonemertean species regarding ancestral larval traits (Thollesson and Norenburg, 2003; Maslakova, 2010b; Andrade et al., 2014; Maslakova and Hiebert, 2014). Therefore, reconstruction of ancestral traits of nervous system development in Nemertea has to rely on the data on paleonemertean and hoplonemertean species and allows the following conclusions to be drawn.

(1) Early, transitory frontal and caudal, flask-shaped 5HT-lir neuron-like signals are present in both hoplonemertean and paleonemertean larvae (Chernyshev and Magarlamov, 2010; Hiebert and Maslakova, 2015; von Döhren, 2016b; this study). It is therefore likely, that a minimum of one caudal and two frontal signals are part of the ground pattern of Nemertea. Similar frontal 5HT-lir neurons are known from larvae of several spiralian species, although their numbers may differ considerably between species (Wanninger, 2008, 2009; Richter et al., 2010; Liang et al., 2023; references therein). These neurons are normally part of the larval apical organ and last for some extended time into larval life before they degenerate at metamorphosis. In Nemertea, with the exception of *Q. stimpsoni*, the early frontal flask-shaped cells become undetectable early on in larval life. It is not clear whether the neurons degenerate or become incorporated into the developing brain ring. In *Q. stimpsoni*, some of the early frontal 5HT-lir neurons are reported to become incorporated into the developing frontal organ of the juvenile (Magarlamov et al., 2020).

(2) Apart from Nemertea, an early transitory caudal neuron is present in annelid, mollusk and platyhelminth species (Voronezhskaya et al., 2003; McDougall et al., 2006; Page, 2006; Rawlinson, 2010; Redl et al., 2014; Starunov et al., 2017; Battonyai et al., 2018; Pavlicek et al., 2018; Yurchenko et al., 2018; Kumar et al., 2020; Fofanova et al., 2021). Although the neuron does not show 5HT-lir in a few investigated species, it is more parsimonious to assume that an early formed caudal 5HT-lir neuron that is transitorily expressed represents an ancestral trait of at least a subgroup of Spiralia that minimally includes the above-mentioned clades.

(3) The brain and the paired, lateral longitudinal, medullary-type nerve cords in Nemertea develop in a frontal to caudal progression without any evident segmental pattern. A frontal brain and a pair of lateral, non-segmented, medullary longitudinal nerve cords has been proposed to most likely represent the ancestral architecture of the so-called central nervous system of Spiralia (Hejnol and Lowe, 2015). In this respect, Nemertea seem to represent the ancestral pattern of structure and position of the nerve cords in Spiralia.

## Data availability statement

The original contributions presented in this study are included in this article/Supplementary material, further inquiries can be directed to the corresponding author.

## Ethics statement

Ethical approval was not required for the study involving animals in accordance with the local legislation and institutional requirements because the animals are lower invertebrates not protected by European legislation.

## Author contributions

JD: Conceptualization, Data curation, Formal analysis, Investigation, Methodology, Project administration, Visualization, Writing – original draft, Writing – review & editing.
