# Supplementary material for: Comparative development of the serotonin- and FMRFamide-immunoreactive components of the nervous system in two distantly related ribbon worm species (Nemertea, Spiralia)
Source: Front Neurosci. 2024 Mar 22;18:1375208. doi: 10.3389/fnins.2024.1375208 (PMC10998470; doi:10.3389/fnins.2024.1375208)
Supplement: Supplementary file 1 [file Table_1.DOCX]

Supplementary Material

**Supplementary Table 1:** Variations of immunostaining protocol, image acquisition and adjustments made with Fiji (ImageJ version 1.54h).

| Specimen shown in | signal enhancer  (incub. time), postfixn 4% PFA in PBS) | blocking:  %PBT + %NGS  (time at RT) | primary AB concentration  (% PBT) | secondary AB incubation time | Z-dimension of layer | projected layers | set contrast | gamma function | rotation |
| --- | --- | --- | --- | --- | --- | --- | --- | --- | --- |
| Fig. 2A | no, no postfix. | 0.1% PBT + 5% NGS (2h) | 1:1000 (0.1%PBT) | 2h | 0.92 µm | 1-35 | 3-57 | 1.5 | -159° |
| Fig. 2B | no, no postfix. | 0.1% PBT + 5% NGS (2h) | 1:750 (0.1%PBT) | 2h | 0.92 µm | 1-34 | 2-95 | 0.6 | -10° |
| Fig. 2C | yes (1h), no postfix. | 0.3% PBT + 10 % NGS (3h) | 1:1000 (0.3%PBT) | 2h | 0.88 µm | 2-45 | 9-88 | 1.2 | -15° |
| Fig. 2D | no, no postfix. | 0.1% PBT + 5% NGS (2h) | 1:1000 (0.1%PBT) | 2h | 0.92 µm | 1-34 | 3-162 | 0.7 | -85° |
| Fig. 3A | yes (1h), no postfix. | 0.3% PBT + 10 % NGS (3h) | 1:1000 (0.3%PBT) | 2h | 0.88 µm | 1-63 | 3-56 | 1.5 | -108° |
| Fig. 3B | yes (1h), no postfix. | 0.3% PBT + 10 % NGS (3h) | 1:1000 (0.3%PBT) | 2h | 0.88 µm | 3-51 | 3-94 | 0.8 | -79° |
| Fig. 3C | yes (1h), no postfix. | 0.3% PBT + 10 % NGS (3h) | 1:1000 (0.3%PBT) | 2h | 0.88 µm | 3-44 | 3-123 | 0.8 | 79° |
| Fig. 3D | no, no postfix. | 0.1% PBT + 5% NGS (2h) | 1:1000 (0.1%PBT) | 2h | 0.92 µm | 1-31 | 4-106 | 1.1 | 19° |
| Fig. 4A | no, no postfix. | 0.1% PBT + 5% NGS (2h) | 1:10 (0.1%PBT) | 2h | 0.92 µm | 1-30 | 2-42 | 1.2 | 21° |
| Fig. 4B | no, no postfix. | 0.1% PBT + 5% NGS (2h) | 1:10 (0.1%PBT) | 2h | 0.92 µm | 1-36 | 2-38 | 1.1 | -101° |
| Fig. 4C | no, no postfix. | 0.1% PBT + 5% NGS (2h) | 1:10 (0.1%PBT) | 2h | 0.92 µm | 3-40 | 5-111 | 0.8 | 123° |
| Fig. 5A | yes (1h10’), no postfix. | 0.2% PBT + 10 % NGS (2h) | 1:1000 (0.2%PBT) | 2h | 0.97 µm | 1-49 | 4-117 | 1.1 | 116° |
| Fig. 5B | yes (1h), no postfix. | 0.2% PBT + 10 % NGS (2h) | 1:1000 (0.2%PBT) | 2h | 0.88 µm | 10-52 | 4-123 | 1.0 | -16° |
| Fig. 5C | yes (1h), no postfix. | 0.2% PBT + 10 % NGS (2h) | 1:1000 (0.2%PBT) | 2h | 0.88 µm | 1-42 | 2-102 | 0.8 | -101° |
| Fig. 5D | yes (1h), postfix. (15’) | 0.2% PBT + 10 % NGS (2h) | 1:1000 (0.2%PBT) | 2.5h | 0.88 µm | 5-63 | 29-2885 | 0.7 | -122° |
| Fig. 5E | yes (1h), postfix. (10’) | 0.2% PBT + 10 % NGS (3h) | 1:1000 (0.2%PBT) | 2.5h | 1.01 µm | 29-37 | 29-1369 | 0.8 | 7° |
| Fig. 6A | yes (1h10’), no postfix. | 0.2% PBT + 10 % NGS (2h) | 1:1000 (0.2%PBT) | 2h | 0.97 µm | 1-50 | 3-48 | 1.2 | 153° |
| Fig. 6B | yes (1h), no postfix. | 0.2% PBT + 10 % NGS (2h) | 1:1000 (0.2%PBT) | 2h | 0.88 µm | 6-47 | 4-83 | 0.8 | -168.5° |
| Fig. 6C | yes (1h), no postfix. | 0.2% PBT + 10 % NGS (2h) | 1:1000 (0.2%PBT) | 2h | 0.88 µm | 1-47 | 4-85 | 0.8 | 150° |
| Fig. 6D | yes (1h), postfix. (15’) | 0.2% PBT + 10 % NGS (2h) | 1:1000 (0.2%PBT) | 2.5h | 0.88 µm | 1-63 | 33-1037 | 1.2 | -99.5° |
| Fig. 6E | yes (1h), postfix. (10’) | 0.2% PBT + 10 % NGS (3h) | 1:1000 (0.2%PBT) | 2.5h | 1.01 µm | 25-33 | 55-1997 | 0.8 | 136.5° |
| Fig. 7A | yes (1h10’), no postfix. | 0.2% PBT + 10 % NGS (2h) | 1:50 (0.2%PBT) | 2h | 0.97 µm | 2-50 | 3-28 | 0.9 | 57° |
| Fig. 7B | yes (1h), no postfix. | 0.2% PBT + 10 % NGS (2h) | 1:50 (0.2%PBT) | 2h | 0.88 µm | 1-43 | 5-35 | 1.1 | 164.5° |
| Fig. 7C | yes (1h), postfix. (15’) | 0.2% PBT + 10 % NGS (2h) | 1:50 (0.2%PBT) | 2.5h | 0.88 µm | 6-51 | 97-1175 | 1.3 | -65° |
